# The roles of liquidity and delay in financial markets based on an optimal forecasting model

**DOI:** 10.1371/journal.pone.0290869

**Published:** 2023-09-01

**Authors:** Guo-Hui Yang, Si-Qi Ma, Xiao-Dong Bian, Jiang-Cheng Li

**Affiliations:** School of Finance, Yunnan University of Finance and Economics, Kunming, P. R. of China; Roma Tre University: Universita degli Studi Roma Tre, ITALY

## Abstract

We investigate the roles of liquidity and delay in financial markets through our proposed optimal forecasting model. The efficiency and liquidity of the financial market are examined using stochastic models that incorporate information delay. Based on machine learning, we estimate the in-sample and out-of-sample forecasting price performances of the six proposed methods using the likelihood function and Bayesian methods, and the out-of-sample prediction performance is compared with the benchmark model ARIMA-GARCH. We discover that the forecasting price performance of the proposed simplified delay stochastic model is superior to that of the benchmark methods by the test methods of a variety of loss function, superior predictive ability test (SPA), Akaike information criterion (AIC), and Bayesian information criterion (BIC). Using data from the Chinese stock market, the best forecasting model assesses the efficiency and liquidity of the financial market while accounting for information delay and trade probability. The rise in trade probability and delay time affects the stability of the return distribution and raises the risk, according to stochastic simulation. The empirical findings show that empirical and best forecasting approaches are compatible, that company size and liquidity (delay time) have an inverse relationship, and that delay time and liquidity have a nonlinear relationship. The most efficient have optimal liquidity.

## 1. Introduction

In recent decades, using statistical physics and complex system methods to study financial systems has become a common phenomenon in the fields of natural science and economics [1, 2]. Researchers have built many macro- and micro-models [1], and deeply analyzed the complex dynamic behaviors and phenomena of financial systems, for instance, critical phase transition behavior [3], power law phenomena and critical behaviors [4], volatility enhanced financial market stability [5], stochastic interaction dynamics of financial systems and their complexity [6], the herd behavior of mutual funds by using complex network method [7], etc. Moreover, traditional financial theory believes that the market is balanced and efficient. For example, Eugene Fama proposed the Efficient Markets Hypothesis (EMH) [8]. However, empirical study has identified many phenomena that defy easy explanation by the EMH, including resonance [9], leptokurtic and fat-tailed phenomena [10], volatility clustering [11], and critical phenomenon [1, 12].

Moreover, EMH is also hard to explain complex capital market instability such as leverage effect and herd effect [13], financial crises [14], market panics [15], and stock market crashes [16–20], etc. These examples illustrate the imperfections and inefficiencies of the capital market, highlighting its tendency towards imbalances and instability, particularly evident during stock market crashes when liquidity becomes scarce, as exemplified by the 1987 stock market crash [21] and the 2008 financial crisis [22]. In the case of severe illiquidity, investors will find it difficult to clear their significant trading positions at market prices, and investors in the capital market will face huge liquidity risks. Therefore, liquidity risk is one of the critical risks that investors pay attention to in stock market trading, and this aspect has also been widely concerned and studied. For instance, Chang et al. found that stock liquidity increases stock price crash risk [23]; Bai et al. constructed a liquidity mismatch index [24]; Gao et al. analyzed the dynamic relationship between internet attention and stock market liquidity [25]. As a result, the prices in the real market cannot fully and immediately reflect all the information available, indicating the presence of information time delay effects. It is important to note that information delay exists and can be observed and validated in various natural and financial systems, such as, forecasting price delay [26], the optimal investment with delay under partial information [27], linear control for synchronization of a fractional-order time-delayed chaotic financial system [28], stability for a novel time-delay financial hyperchaotic system by adaptive periodically intermittent linear control [29], information delay restraining the herd behavior of stock market [30], etc. The ARCH model and a series of derived models can also confirm the existence of the time delay effect [31–33]. Hence, it is necessary to introduce information delay to describe complex financial systems.

We can uncover various financial anomalies by analyzing market disequilibrium, equilibrium, and other investigations. We also focus on examining liquidity and time delay as separate factors.

Therefore, our research approach involves studying financial markets from information time delay and liquidity, building upon existing research in this area. Using our proposed forecasting model, we investigate the roles played by liquidity and delay in financial markets. To achieve this, we introduce a delay time component to construct a delay model and proceed to compare its performance in both in-sample and out-of-sample datasets. Finally, we assess market efficiency by considering the impact of delay time, aiming further to explore the effects of market efficiency and liquidity. The differences and innovations of this paper are as follows. (1) Non-equilibrium statistical physics [34] is used to analyze market disequilibrium. To describe market efficiency, information delay [26] is introduced. (2) The dynamic prediction in our study is generated using a machine learning algorithm [35], which utilizes samples from both the training set and the testing set. To ensure the accuracy and reliability of the prediction, we incorporate a rolling cycle [36], which allows the algorithm to continuously update and adapt to new data as it becomes available. This iterative process enhances the model’s ability to capture and incorporate the changing dynamics of the financial market, resulting in more accurate and up-to-date predictions. (3) The multi-loss function is used to provide the prediction ability and test six techniques. To evaluate the predictive power of our suggested models, we introduce a range of loss functions, the Superior Predictive Ability Test (SPA) [35–37], the Akaike Information Criterion (AIC) [38, 39], and the Bayesian Information Criterion (BIC) [40, 41]. (4)The likelihood function [42] and Bayesian [43] methods of the proposed delay model are presented, and empirical studies are also discussed. The rest of this paper is organized as follows. Section 2 presents the models and methods for efficiency and stability. Section 3 discusses forecasting evaluation. Section 4 shows Forecasting empirical comparison. Section 5 shows empirical analysis of efficiency and liquidity. A brief discussion is given in section 6.

## 2. Models and method

### 2.1 Delay model for efficiency and liquidity

The stochastic model is a standard macro model that characterizes the dynamics of financial market price dynamics, such as the ARCH, GARCH, and Heston models. It is difficult to estimate the price volatility in various models. Such as Li et al. [37], Zhou et al. [44], Feng et al. [45], Zheng et al. [46], Gontis et al. [47] and many other scholars consider using the absolute value of return instead of price volatility, which can also better fit the actual data. In the realm of financial market analysis, the utilization of high-frequency data has opened up new possibilities for measuring market volatility. One commonly employed approach is realized volatility, which offers a straightforward method for calculating market volatility [48]. Additionally, Feng et al. [45] proposed an agent-based model (ABM) and stochastic Model (SM) to investigate market dynamics and behavior. Subsequently, Li et al. [37] conducted prediction analysis on these models and introduced the Simplified Stochastic Model (SSM). Their findings demonstrated that SSM consistently yielded accurate predictions. When only simple price series data is available, using the absolute value of price return as a measure of volatility is a convenient and effective method. Based on the methods in Refs. [37, 44, 45], the delay stochastic model (DSM) is proposed by comparing it with the delay agent-based model (DABM) [44]:

$$\begin{array}{ccc} r_{t} & = & \sigma_{r}\left(t\right)\eta_{t},\\ \sigma_{r}^{2}\left(t\right) & \equiv & E\left(r_{t}^{2}|r_{t-\tau }\right)=2c_{t}pr_{t-\tau }^{2}. \end{array}$$ (1)

*p* is the trading probability derived from the delay agent-based model (DABM) [44]. Considering micro-trading behavior, each day a trading decision *ψ*_*i*_(*t*) is made by each agent *i*

$$\begin{array}{c} \psi_{i}\left(t\right)\equiv \{\begin{array}{cc} -1 & with\;tradingprobability\;p\;\Rightarrow \;sell;\\ 0 & with\;tradingprobability\;1-2p\;\Rightarrow \;hold;\\ 1 & with\;tradingprobability\;p\;\Rightarrow \;buy. \end{array} \end{array}$$

Considering the random fluctuations of the investor’s point of view clustering *c*_*t*_, based on the description of Step (iii) in the previous part of the micro model, we get the following equation.

$$\begin{array}{ccc} c_{t} & = & (n_{0}/|r_{t-\tau }{\left|\right)}^{\varpi }+\sigma_{c}\left(t\right)\xi_{t},\\ \sigma_{c}^{2}\left(t\right) & = & \beta n_{0}/\left|r_{t-\tau }\right|. \end{array}$$

Finally, we can get our delay stochastic model

$$\begin{array}{ccc} r_{t} & = & \sqrt{2c_{t}p}\left|r_{t-\tau }\right|\eta_{t},\\ c_{t} & = & (n_{0}/|r_{t-\tau }{\left|\right)}^{\varpi }+\sqrt{\beta n_{0}/\left|r_{t-\tau }\right|}\xi_{t}, \end{array}$$ (2)

where *η*_*t*_ and *ξ*_*t*_ are the random variables with zero mean and unit variance, their correlation coefficient is *ρ*, and their distributions are determined by step 3 of the above section, 1 ≤ |*r*_*t*_| ≤ *n*_0_ and 1 ≤ *c*_*t*_ ≤ *n*_0_. When the delay parameter *τ* is set to 1, the delay models discussed in references [37, 45] revert to their original forms. These references primarily utilize information from the previous period, whether one day or one trading cycle, to predict future market dynamics. In essence, these models can be classified as period one delay models. In order to better calculate the delay time, considering that $E\left(c_{t}\right)=(n_{0}/|r_{t-\tau }{\left|\right)}^{\varpi }$, so DSM can be simplified to

$$\begin{array}{c} r_{t}=\sqrt{2n_{0}^{\varpi }p{\left|r_{t-\tau }\right|}^{2{-}^{\varpi }}}\eta_{t}. \end{array}$$

If ϖ=1 is taken from Refs. [37, 45], the simplified delay stochastic model (SDSM) can be obtained.

$$\begin{array}{c} r_{t}=\sqrt{2n_{0}p\left|r_{t-\tau }\right|}\eta_{t}. \end{array}$$

According to Fama’s Efficient Market Hypothesis (EMH), the efficiency of a market increases with faster information response. Hence, the information delay time can serve as a measure of market efficiency. A larger delay time indicates lower market efficiency, while a smaller delay time suggests a more efficient market.

### 2.2 Likelihood function method

Considering *n*_0_ = 2^10^ and ϖ=1 the same as in reference [45], the unknown parameters can be replaced with **θ** = (*p*, *τ*). The likelihood function estimation with the sample *r* = (*r*_0_, *r*_1_, …, *r*_*n*_) can be obtained as

$$\begin{array}{c} {\hat{\theta }}_{MLE}=\underset{\theta }{\text{arg}\;\text{max}}\left\{L\left(\theta \right)\right\}, \end{array}$$ (3)

where

$$\begin{array}{ccc} L(\theta ) & = & \sum_{s=1}^{n}\;\text{log}\;P\left(r_{s},c_{s}|\theta \right)\\ & = & -\sum_{t=\tau }^{n}\{\frac{1}{2}ln(2n_{0}p|r_{t-\tau }\left|\right)+\frac{r_{t}^{2}}{4n_{0}p\left|r_{t-\tau }\right|}\}. \end{array}$$ (4)

For DSSM, let Δ|*r*_*t*−*τ*_| = |*r*_*t*−*τ*_| − |*r*_*t*−*τ*−1_| and Δ^2^|*r*_*t*−*τ*_| = Δ|*r*_*t* − *τ*_| − Δ|*r*_*t*−*τ*−1_|, based on $\frac{\partial L(\theta )}{\partial p}=0$ and $\frac{\partial L(\theta )}{\partial \tau }=0$, the following equations can be obtained

$$\begin{array}{ccc} & & p=\frac{1}{n-\tau }\sum_{t=\tau }^{n}\frac{r_{t}^{2}}{2n_{0}\left|r_{t-\tau }\right|},\\ & & \sum_{t=\tau }^{n}\left[\frac{\left(\Delta \right|r_{t-\tau }{\left|\right)}^{2}}{|r_{t-\tau }{|}^{2}}-\frac{\Delta^{2}\left|r_{t-\tau }\right|}{|r_{t-\tau }|}+\frac{r_{t}^{2}\Delta^{2}\left|r_{t-\tau }\right|}{2n_{0}p{\left|r_{t-\tau }\right|}^{2}}-\frac{r_{t}^{2}\left(\Delta \right|r_{t-\tau }{\left|\right)}^{2}}{4n_{0}p{\left|r_{t-\tau }\right|}^{3}}\right]=0. \end{array}$$ (5)

The Eq (5) only needs *r*_*t*_, which is easy to obtain data and simple to calculate, providing a relatively fast method to estimate the delay of market information. Based on the Eq (4), the annealing algorithm [49, 50] can be used to obtain the model parameters.

### 2.3 Bayesian estimation method

For observation samples *r*_obs_ [*n* observations *r*_obs_ = (*r*_1_, *r*_2_, …, *r*_*n*_)], the posterior distribution of the model can be obtained. We can get the equation as follow.

$$\begin{array}{c} \pi \left(\theta |r_{\text{obs}}\right)\;\propto \;\pi \left(\theta \right)\prod_{i=1}^{n}P\left(r_{t}|\theta ,c_{t}\right). \end{array}$$ (6)

Based on the DSSM model settings in section 2, we give the prior distribution of unknown parameters of the model *P*(*θ*) = **P**(**p**, *τ*) = **P**(**p**|*τ*)**P**(*τ*)

$$p∼IGa\left(a_{D0},b_{D0}\right),\tau ∼U\left(0,u_{D0}\right).$$

We can obtain a posteriori distribution of parameters based on observed samples

$$\begin{array}{ccc} \pi (\theta |r_{\text{obs}}) & \propto & \pi \left(\theta \right)\prod_{i=1}^{n}P\left(r_{t}|\theta ,c_{t}\right)\\ & \propto & \pi \left(\theta \right)p^{-n/2}\\ & & exp\left\{-\frac{1}{p}\sum_{t=1}^{n}\frac{r_{t}^{2}}{4n_{0}\left|r_{t-\tau }\right|}\right\}\prod_{i=1}^{n}\frac{1}{\sqrt{2n_{0}p\left|r_{t-\tau }\right|}}. \end{array}$$ (7)

Based on the previous likelihood and prior distribution, the delay time can be obtained by the MH algorithm

$$\begin{array}{ccc} \tau_{i}|p,c_{t},x_{t} & ∼ & U(0,2\tau_{i-1}), \end{array}$$ (8)

and

$$\text{min}\{1,\frac{\pi \left(\tau^{*}|x_{\text{obs}}\right)q\left(\tau^{i}|\tau^{*}\right)}{\pi \left(\tau^{i}|x_{\text{obs}}\right)q\left(\tau^{*}|\tau^{i}\right)}\}.$$

We can get the unknown parameter *p* posterior sampling calculation formula

$$\begin{array}{ccc} p|\tau ,c_{t},x_{t} & ∼ & IGa(a_{p1},b_{p1}), \end{array}$$ (9)

where

$$\begin{array}{ccc} a_{p1} & = & a_{p0}-1+\frac{n}{2},b_{p1}=b_{p0}+\sum_{t=1}^{n}\frac{r_{t}^{2}}{4n_{0}\left|r_{t-\tau }\right|}. \end{array}$$

*a*_*p*0_,*b*_*p*0_ and *u*_*D*0_ are hyper parameters. In comparison with the likelihood method, in order to eliminate the influence of prior distribution, no information prior distribution is adopted in the Bayesian estimation method.

## 3. Forecasting evaluation

### 3.1 Collection of models and methods

In the case of only income samples, the proposed model consists of two estimation methods and is compared with the existing benchmark methods without delay. There are six methods in total as shown in the Table 1. M1 and M2 are our proposed methods. We introduce information delay time, propose a new stochastic delay model, and construct M1 and M2 methods. In order to evaluate the prediction performance of M1 and M2 methods, empirical comparisons have been made with other benchmark models M3, M4, M5, and M6 obtained from the Ref. [37].

**Table 1:** Collection of models and methods.

Short Name	Combination method
M1	DSSM model + Likelihood method
M2	DSSM model + Bayesian method
M3	SSM model + Likelihood method
M4	SSM model + Bayesian method
M5	ABM model + Bayesian method
M6	SM model + Bayesian method

### 3.2 Dynamic forecasting and evaluation

To evaluate the forecasting performance of our proposed delay model, machine learning was employed to evaluate the in-sample and out-sample forecasting performance of the proposed methods. We divide the samples into “estimate sample” and “forecast sample” two parts [35, 37]. “Forecast Sample” is fixed as the data containing *M* trading days, and the “Estimated Sample” is fixed as the data containing *H* trading days before “Forecast Sample”. The parameters of the proposed six methods are estimated from “Estimated Sample”, and the forecasting time series are calculated by stochastic simulation with the obtained parameters, as follows.

- Step 1: The data of *H* trading days before each prediction sample is selected as an estimation sample to estimate the parameters of the proposed model set.
- Step 2: Based on the estimated parameters, the price and return of the *H* + 1 trading day are randomly simulated as the forecast data.
- Step 3: From the first prediction sample to *M* prediction samples, repeat the first step process to obtain the forecasting time series.

After the prediction time series is obtained, to further evaluate and test the prediction performance of the six methods, we conduct analysis based on the loss functions and test methods. Many studies [35–37] have found that if a single loss function estimation model is used to predict performance, its accuracy may be insufficient. Therefore, five commonly used loss functions are used to evaluate the performance of the proposed methods. The five kinds of loss functions are defined as follows:

$$\begin{array}{ccc} Loss_{1} & : & MAE=M^{-1}\sum_{H+1}^{H+M}\left|S_{m}-S_{m}^{\prime }\right|, \end{array}$$

$$\begin{array}{ccc} Loss_{2} & : & MSE=M^{-1}\sum_{H+1}^{H+M}{\left(S_{m}-S_{m}^{\prime }\right)}^{2}, \end{array}$$

$$\begin{array}{ccc} Loss_{3} & : & MAPE=M^{-1}\sum_{H+1}^{H+M}\left|\frac{S_{m}-S_{m}^{\prime }}{S_{m}}\right|, \end{array}$$

$$\begin{array}{ccc} Loss_{4} & : & MSPE=M^{-1}\sum_{H+1}^{H+M}{\left[\frac{S_{m}-S_{m}^{\prime }}{S_{m}}\right]}^{2}, \end{array}$$

$$\begin{array}{ccc} Loss_{5} & : & MSigmaE=M^{-1}\sum_{H+1}^{H+M}{\left[\frac{S_{m}-S_{m}^{\prime }}{\sigma (S_{m})}\right]}^{2}, \end{array}$$

here, *M* is the length of the prediction set. However, comparing the forecasting performance of the six methods only by the loss functions is not robust. Therefore, out-of-sample dynamic forecasting performance is further examined based on the superior predictive ability test (SPA) [35–37], Akaike information criterion (AIC) [38, 39] and Bayesian Information Criterion (BIC) [40, 41].

## 4. Forecasting empirical comparison

### 4.1 Data

We discuss the market’s overall stability and conduct empirical research on the model using the CSI 300 index to replace the market portfolio. The data source is the CSMAR database from the start of the CSI 300 index to March 18, 2022 (China Stock Market & Accounting Research Databases). The statistical characteristics of index prices, returns and absolute returns are further calculated as shown in the Table 2. The stock price return (return in Table 2) can be written as [51]: *return* = *log*(*P*_*i*_/*P*_*i*−1_), where *log*(*P*_*i*_) is logarithmic price (lnprice in Table 2) at the *i*th time point for *i* = 1, 2, 3, ⋯.

**Table 2:** Table of statistical characteristics of real sample of CSI300 data.

–	lnprice	return	Absolute value of return
mean	7.9751	0.0004	0.0116
min	6.7069	-0.0970	0.0000
max	8.6788	0.0893	0.0970
SD	0.4387	0.0168	0.0122
skewness	-1.1538	-0.5171	2.2865
kurtosis	1.0408	3.9992	7.4512
Q(5)	20729.00	18.90	841.07
Q(10)	41147.42	38.26	1665.25
Q(20)	80970.72	63.45	2893.91
J-B	1114.43	2963.07	13282.54
ADF	-2.4116	-14.2344	-8.0932

The descriptive statistical results of Table 2 show that the return data is obviously “leptokurtic and fat-tailed” (excess coefficient). The absolute value of the return is also used to measure one of the essential indicators of price volatility [52]. The Jarque-Bera statistic illustrates that the CSI 300 index fluctuates wildly, far beyond the range assumed by the normal distribution; this also shows that the prices and fluctuations of the CSI 300 index have significant persistence or long-term memory characteristics. The ADF unit root test shows that all returns reject the null hypothesis of unit root significantly. The benefits are smooth, allowing for direct analysis and quantitative modeling. According to the study, financial market volatility increases after the onset of COVID-19, making model prediction challenging. A black swan event, COVID-19 has significant effects on the world economy. With concerns about the coronavirus, American equities eventually concluded the longest bull market in history. Financial markets in China were also severely impacted. In order to focus on the impact of information delay and flow on the market, modeling, and empirical analysis, to eliminate the impact of abnormal events, we mainly consider the data after the impact of the COVID-19 epidemic. Consequently, we primarily chose the post-COVID-19 data as the prediction set from January 3, 2020, to March 18, 2022, to assess the model’s prediction performance. Considering the information delay amount *τ* in the calculation of the model, the first 100 days were analyzed as the empty amount of *τ*, which also avoided the impact of COVID-19 in the early stage.

### 4.2 Forecasting empirical comparison for in-sample test

**Fig 1:**
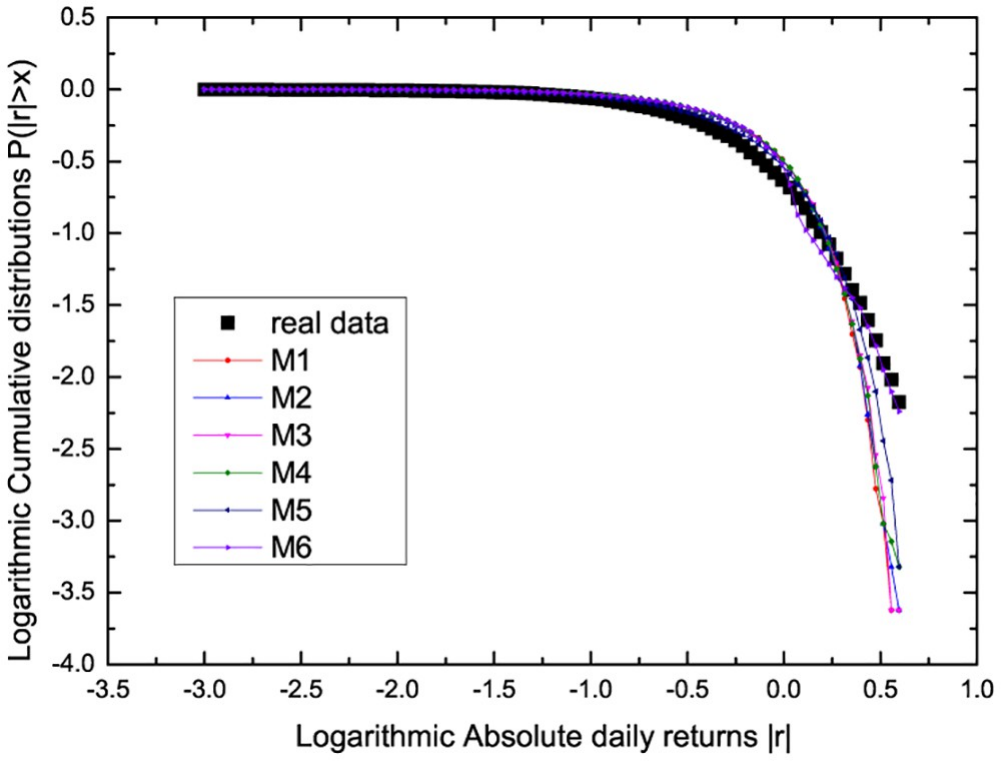
Comparison of the distributional properties of simulations with empirical results of CSI300 for the parameter set.

The DSSM model’s parameter estimation is calculated using the likelihood function method with CSI 300 index data in section 2, and the benchmark models in Ref. [37] are also presented. Based on the estimated results, stochastic simulations are performed for the six proposed models. The comparison of the distributional properties of simulations with empirical results of CSI300 for the parameter set is calculated and presented in Fig 1. It can be found that the actual data results are in good agreement with the simulation results of the proposed six methods.

To further test the goodness of fit for in-sample, the AIC and BIC values of the six methods for CSI300 based on the estimated parameters are calculated as shown in Fig 1. The test results are presented in Table 3. Based on the minimum information principle, the AIC and BIC values are the M2 method, followed by M1. For a given CSI300, the M2 method performed best, followed by M1. This shows that our proposed model is superior to the original model. In other words, introducing delay time can enhance the in-sample prediction performance of the model. This implies a time-delay effect in financial markets. Therefore, we can further analyze the impact of market delay and liquidity on market efficiency through M1 or M2 methods.

**Table 3:** AIC and BIC values of the 6 models with CSI300 for in-sample.

	M1	M2	M3	M4	M5	M6
AIC	-34135.5	-34140.7	-34113.7	-34134.0	-33991.0	-33079.5
BIC	-34122.8	-34128.1	-34101.0	-34121.3	-33978.3	-33073.2

### 4.3 Forecasting empirical comparison for out-sample test

Next, we further analyze and evaluate the out-of-sample prediction performance of the six methods, and compare the out-of-sample forecasting performance of proposed methods for real CSI300. The prediction set is set to post-COVID-19 data from January 3, 2020, to March 18, 2022. In order to eliminate the influence of prior distribution, the prior distribution adopts no information prior in the method of Bayesian estimation.

First, to discuss the influence of a small training set on the prediction, we calculate the loss functions of the six proposed methods under different training lengths *H*. The result is presented in the Table 4. For most loss function cases, the range from *H* = 100 to *H* = 300 shows that of all the loss functions, *M*1 is the smallest, followed by *M*2. In other words, the out-of-sample predictive performance of *M*1 and *M*2 is better than that of other methods. M1 and M2 are our proposed delay model’s likelihood estimation and Bayesian estimation methods. This shows that introducing time delay can enhance the out-of-sample prediction performance of the model.

**Table 4:** Five loss functions of logarithmic price out-of-sample prediction of the 7 models with CSI300 for different *H*.

		MAE	MSE	MAPE	MSPE	MSsigmaE
H = 100	M1	0.00973	0.0001776	0.00115	0.0000025	1.08461
	M2	0.00973	0.0001785	0.00115	0.0000025	1.09472
	M3	0.00975	0.0001788	0.00116	0.0000025	1.09423
	M4	0.00981	0.0001805	0.00116	0.0000025	1.10642
	M5	0.01821	0.0005533	0.00215	0.0000077	2.92454
	M6	0.00990	0.0001852	0.00117	0.0000026	1.13692
	ARIMA-GARCH	0.01159	0.00028	0.00145	0.0000044	0.97667
H = 300	M1	0.00975	0.0001784	0.00115	0.0000025	1.09200
	M2	0.00973	0.0001783	0.00115	0.0000025	1.09218
	M3	0.00974	0.0001786	0.00115	0.0000025	1.09381
	M4	0.00974	0.0001786	0.00115	0.0000025	1.09466
	M5	0.01019	0.0001954	0.00121	0.0000028	1.22997
	M6	0.00976	0.0001786	0.00116	0.0000025	1.09382
	ARIMA-GARCH	0.01187	0.000298	0.00148	0.0000046	0.95953

Comparison with benchmark models is a common approach in forecasting research, such as, a forecasting tool based on the ARIMAX and Cox-Ingersoll-Ross (CIR) models [53], the interest rates forecasting via a modified version of the CIR model [54, 55]. Hence, we contrast the six suggested strategies with the ARIMA-GARCH model in order to further compare them with the benchmark model. Hence, we contrast the six suggested strategies with the ARIMA-GARCH model to compare them with the benchmark model further. A more popular volatility prediction model is ARIMA-GARCH. We used the fundamental ARIMA-GARCH, and the estimated outcomes are displayed in Table 4. The lagged order of ARIMA-GARCH is determined by the AIC criterion concerning the idea in Ref. [54]. The suggested six models are found to perform better than the ARIMA-GARCH-predicted results under various time Periods according to MAE, MSE, MAPE, and MSPE results. According to the MSsigmaE results, ARIMA-GARCH was superior. The majority of the loss functions, as can be seen from the many loss functions we frequently employ, indicate that the six models are superior to ARIMA-GARCH.

In order to further verify the conclusions obtained by the loss function, we adopt the SPA test for analysis. SPA test results obtained after 10,000 bootstrap simulations are presented in Table 5. The first column represents the five loss functions; the second column is the model’s name selected as the base model. The number in the table is the p-value of the SPA test. The higher the *p* value is (the closer it is to 1), the higher the prediction accuracy of the basic model will be. Conversely, if the p-value is smaller, it is reasonable to believe that the performance of the underlying model is inferior to that of the comparison model under investigation. As can be seen from the empirical results in Table 5: (1) SPA of the five loss functions shows that *M*1 has better prediction performance than the other five methods; (2) SPA of *MAE*, *MSE* and *MAPE* loss functions all show that M2 had better prediction performance than *M*3, *M*4, *M*5 and *M*6. This also indicates that the out-of-sample prediction performance of the delay model established by introducing delay time is better and further demonstrates the hypothesis of the information delay effect in the model proposed by us.

**Table 5:** SPA test of 8 methods for return prediction.

Loss	Basic model	Comparative model					
		M1	M2	M3	M4	M5	M6
MAE	M1	0.0000	0.5088	0.7645	0.9213	1.0000	0.7949
	M2	0.4927	0.0000	0.7130	0.8922	1.0000	0.8200
	M3	0.2343	0.2843	0.0000	0.8684	1.0000	0.7640
	M4	0.0804	0.1095	0.1246	0.0000	1.0000	0.6647
	M5	0.0000	0.0000	0.0000	0.0000	0.0000	0.0000
	M6	0.1980	0.1771	0.2406	0.3447	1.0000	0.0000
MSE	M1	0.0000	0.7411	0.8838	0.9527	1.0000	0.9026
	M2	0.2604	0.0000	0.5933	0.8382	1.0000	0.9112
	M3	0.1179	0.4110	0.0000	0.8781	1.0000	0.8634
	M4	0.0460	0.1590	0.1133	0.0000	1.0000	0.7788
	M5	0.0000	0.0000	0.0000	0.0000	0.0000	0.0000
	M6	0.0970	0.0938	0.1375	0.2231	1.0000	0.0000
MAPE	M1	0.0000	0.5098	0.7705	0.9213	1.0000	0.7882
	M2	0.5038	0.0000	0.7037	0.8857	1.0000	0.8135
	M3	0.2394	0.2815	0.0000	0.8775	1.0000	0.7663
	M4	0.0816	0.1116	0.1337	0.0000	1.0000	0.6597
	M5	0.0000	0.0000	0.0000	0.0000	0.0000	0.0000
	M6	0.2008	0.1881	0.2336	0.3445	1.0000	0.0000
MSPE	M1	0.0000	0.7751	0.8728	0.9251	0.9996	0.8628
	M2	0.2181	0.0000	0.4882	0.7717	0.9998	0.8567
	M3	0.1291	0.5248	0.0000	0.8463	0.9997	0.8258
	M4	0.0720	0.2275	0.1564	0.0000	0.9997	0.7332
	M5	0.0003	0.0002	0.0002	0.0007	0.0000	0.0003
	M6	0.1433	0.1406	0.1732	0.2641	0.9992	0.0000
MSigmaE	M1	0.0000	0.7767	0.8697	0.9285	0.9998	0.8613
	M2	0.2150	0.0000	0.4853	0.7690	0.9999	0.8609
	M3	0.1322	0.5088	0.0000	0.8367	0.9996	0.8314
	M4	0.0730	0.2293	0.1652	0.0000	0.9999	0.7302
	M5	0.0004	0.0005	0.0002	0.0002	0.0000	0.0005
	M6	0.1394	0.1408	0.1719	0.2686	0.9992	0.0000

Note: The figure in the table is the SPA test *p* value after 10,000 Bootstrap simulation. The larger the *p* value, the better the performance of the basic model compared with the comparison model investigated.

We used another dataset for further comparison to further compare the rationality and stability of the proposed method. We selected 5896 samples of S&P 500 data from January 4, 2020, to May 31, 2023. The data source is also the CSMAR database (China Stock Market & Accounting Research Databases). Use the same calculation method as Table 4. Similarly, the prediction results of the six proposed combined models and the ARIMA-GARCH model are presented in Table 6. It can be seen that the optimal M1 and M2 models found above are still superior to the other four models and ARIMA-GARCH. Of course, ARIMA-GARCH is also an excellent model, with better predictive performance than M5 and M6, and is also very close to other models. Short, M1 and M2 are relatively good models, which describe the information time delay and liquidity in the market and can be used for further analysis of the delay and liquidity problems in the stock market.

**Table 6:** Five loss functions of logarithmic price out-of-sample prediction of the 7 models with the S&P 500 for *H* = 100.

	MAE	MSE	MAPE	MSPE	MSsigmaE
M1	0.00355	0.0000291	0.00111	0.000003	0.92276
M2	0.00353	0.0000291	0.00111	0.000003	0.92132
M3	0.00355	0.0000291	0.00111	0.000003	0.91796
M4	0.00355	0.0000292	0.00111	0.000003	0.92338
M5	0.00582	0.0000674	0.00182	0.000007	2.14177
M6	0.00410	0.0000370	0.00129	0.000004	1.20356
ARIMA-GARCH	0.00357	0.0000301	0.00112	0.000003	0.96400

## 5. Discussion of delay (efficiency) and liquidity

### 5.1 Simulation of delay and liquidity

**Fig 2:**
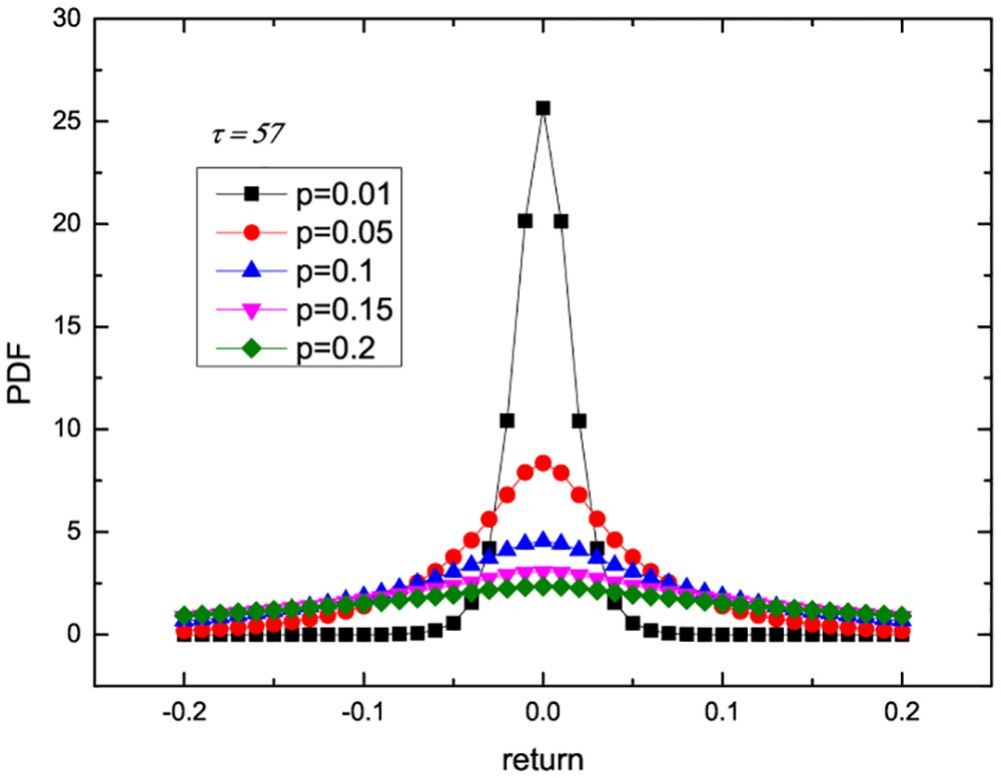
PDF versus trading probability *p* with *τ* = 57.

**Fig 3:**
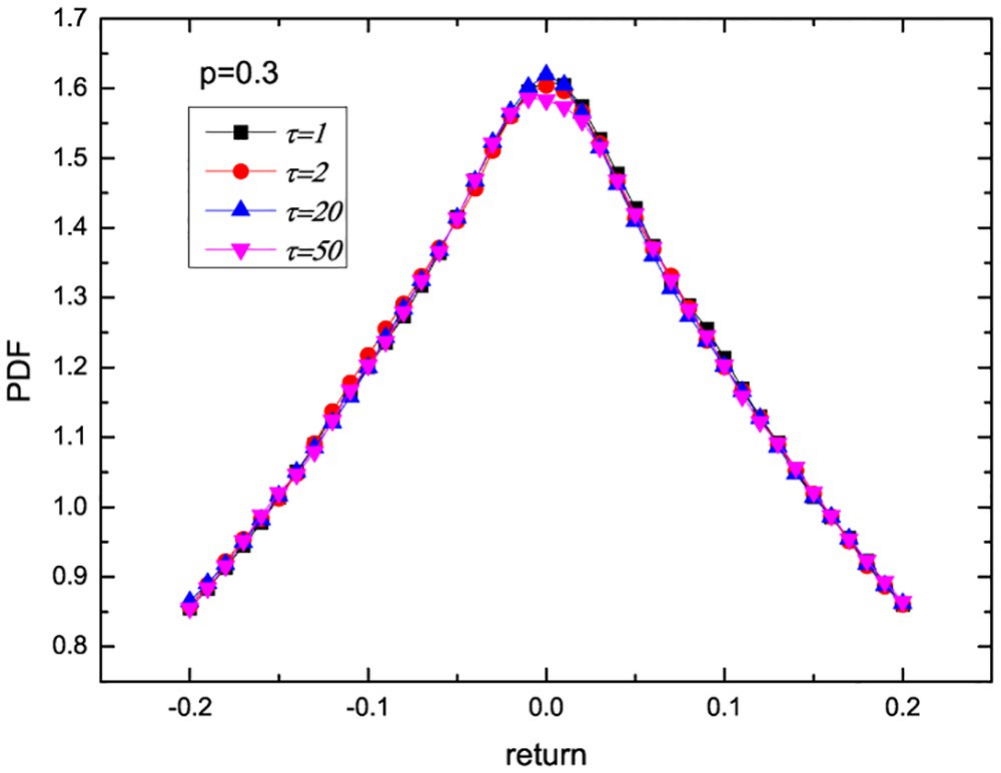
PDF versus delay time *τ* with *p* = 0.3.

Next, based on the optimal forecasting model in the previous part, we further analyze the role of liquidity and delay in financial markets. According to the fitting of Fig 1, the parameters of M1 are estimated as $\hat{p}=0.03262$, and the delay time is integer as $\hat{\tau }=57$. Based on the estimated parameters, we carry out stochastic simulations and calculate the probability distribution function (PDF) as a function of the trading probability and delay time. The results are presented in Figs 2 and 3. Fig 2 shows the PDF versus trading probability *p* when *τ* = 57. It can be found that with the increase of trading probability *p*, the peak value of PDF decreases monotonically. This also implies that the enhancement of liquidity weakens the stability of return distribution and increases the risk. When the trading probability is small, the impact of apparent delay time on the market is almost invisible. When the trading probability *p* = 0.3 is relatively large, Fig 3 shows the PDF versus delay time. We can see that as the delay time *τ* increases, the peak value of PDF decreases. This also implies that the enhancement of information delay (the reduction of efficiency) weakens the stability of income distribution and increases the risk.

### 5.2 Empirical analysis of comparison of delay and trading probability

It can be seen that M1 exhibits strong predictive performance from the forecasting empirical comparison for both the in-sample and out-of-sample tests mentioned above. As a result, we conduct a practical examination of efficiency and liquidity based on M1 and the stock market in the following empirical comparison. To determine the introduction p and delay times of the stock in the market, we employ the approach in the M1 model. Then do an empirical comparative analysis using actual market stock data. A thorough examination of the market’s effects of liquidity and latency.

Due to the large fluctuations in 2015, the data space for apparent extreme financial volatility is eliminated. Data selection, we select all A shares of Shanghai and Shenzhen in the past five years to conduct research. Considering data integrity, we selected stocks with complete data from 2014 to 2019 for analysis. We chose 2,417 stocks from January 1, 2014, to December 31, 2019. The data source is the CSMAR database (China Stock Market & Accounting Research Databases). The sampling frequency is daily.

We calculate the trade probability *p* and *tau* for each stock using the prior DSSM. We also examine the DSSM model’s rationality with other benchmark models and pertinent real-world data to do so. This study reveals that market efficiency is somewhat characterized by the information delay *tau*. In this regard, we can compare the efficiency index of other models with *τ* in DSSM. We introduce variance ratios [56, 57], an efficiency index in another benchmark model, to compare with information delay *τ* in DSSM. Second, the turnover rate of market transactions will unavoidably change if the transaction launch in DSSM is reflected in market transactions. As a result, we present a contrast between the stock market’s turnover rate and the trading probability *p* in the DSSM.

Then based on the daily average of the company size of individual stocks in the period time (January 1, 2016-December 31, 2019), we divide it into ten groups, with the first group having the smallest company size and the tenth group having the largest company size.

**Fig 4:**
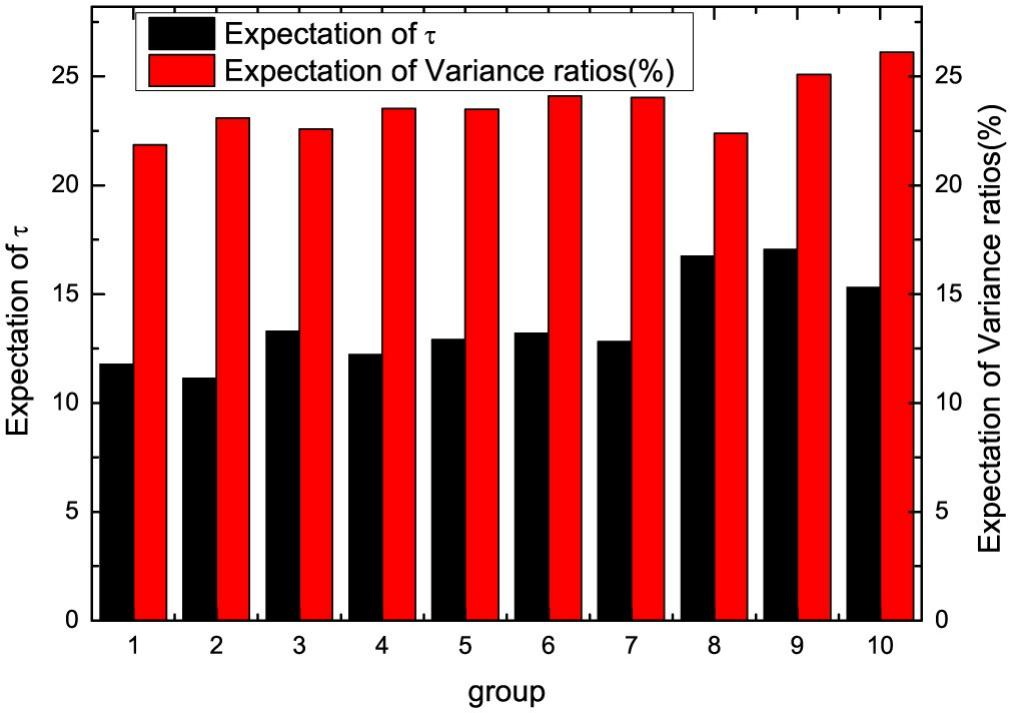
Comparison of the expectation of proposed method *τ* and variance ratios.

**Fig 5:**
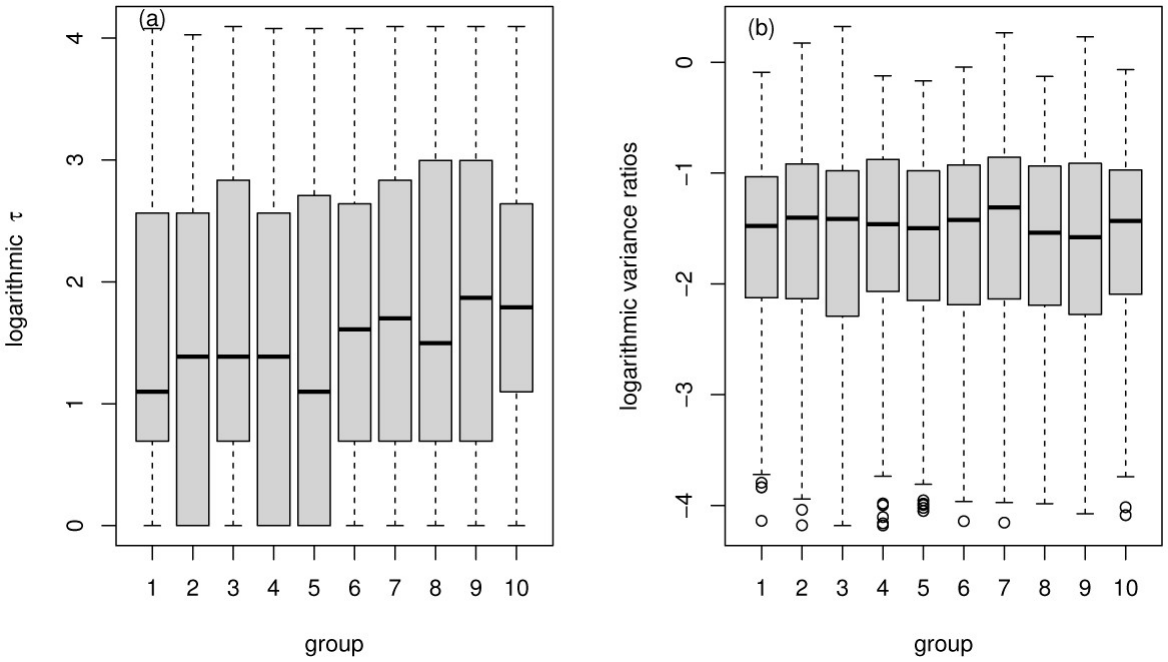
Comparison of the efficiency measure between the proposed method *τ* and variance ratios.

The statistical characteristics of estimated *τ* and variance ratios are obtained in each group. The expectations of the proposed method *τ* and variance ratios are presented in Fig 4. Firstly, we can find that as the company size increases, expectations of *τ* and variance ratios both show an increasing characteristic, which also implies that the market efficiency of stocks with large market capitalization may be lower than that of small market capitalization during this time period. The behavioral characteristics of the two methods are also relatively consistent. For further analysis, we present a box chart of *τ* and variance ratios for company size grouping in Fig 5. From Fig 5, it can be observed that both *τ* and variance ratios also show an overall increasing characteristic with the increase in company size. Of course, we can find that outliers in proposed models are slightly larger than variance ratios. On the one hand, this may be caused by the non-normal characteristics of the data itself. On the other hand, we use the simple DSSM estimation method to get *τ*. We can use more accurate estimation methods to improve accuracy. However, the incremental characteristics between different groups can be observed clearly by the characteristics of the box diagram given by the proposed method, which is convenient for distinguishing differences in efficiency.

**Fig 6:**
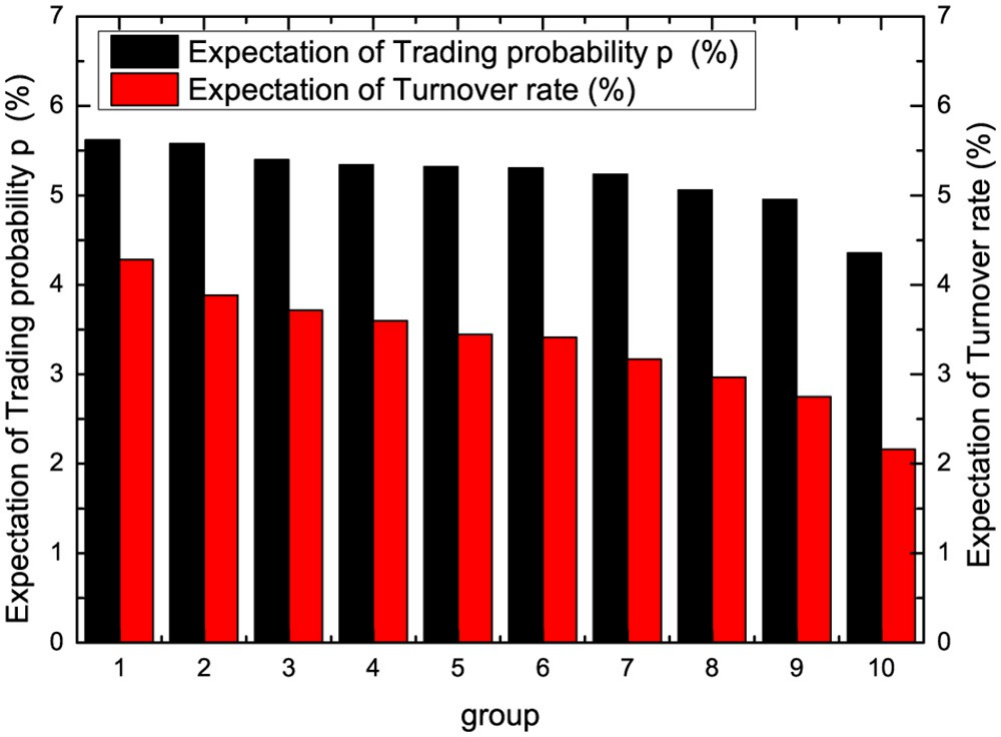
Comparison of the expectations of trading probability and turnover rate.

**Fig 7:**
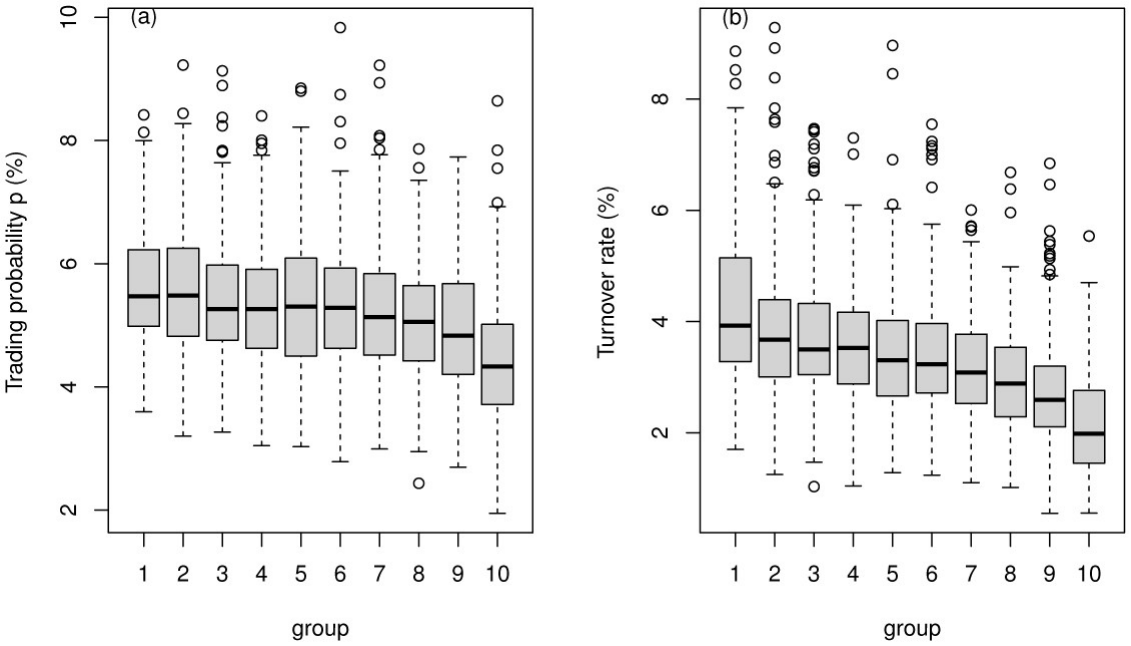
Comparison of the trading probability and turnover rate.

For the measurement of market transaction probability, this paper proposes *p* to measure the market transaction probability. We can adopt the turnover rate to measure the transaction probability in the actual market. There are divided into 10 groups based on the value of the circulating stock market. Then the trading probability and turnover rate expectations are presented as a company size grouping function in Fig 6. Firstly, we can find that the expectations of trading probability and turnover rate both show decreasing characteristics as the company size increases, which also implies that the trading frequency of large company size stocks may be lower than that of small company size during this time period. Secondly, the behavioral characteristics between *p* and turnover rate are also relatively consistent. For further analysis, we present a box plot of trading probability and turnover rate for company size groupings in Fig 7. From the figure, it can be observed that the trading probability and turnover rate also show an overall decreasing characteristic as the company size increases. At the same time, there is not much difference between the two outliers. From the above Figs 4–7, it can be seen that the overall proposed model is more consistent with the actual market.

### 5.3 Empirical analysis of delay (efficiency) and liquidity

**Fig 8:**
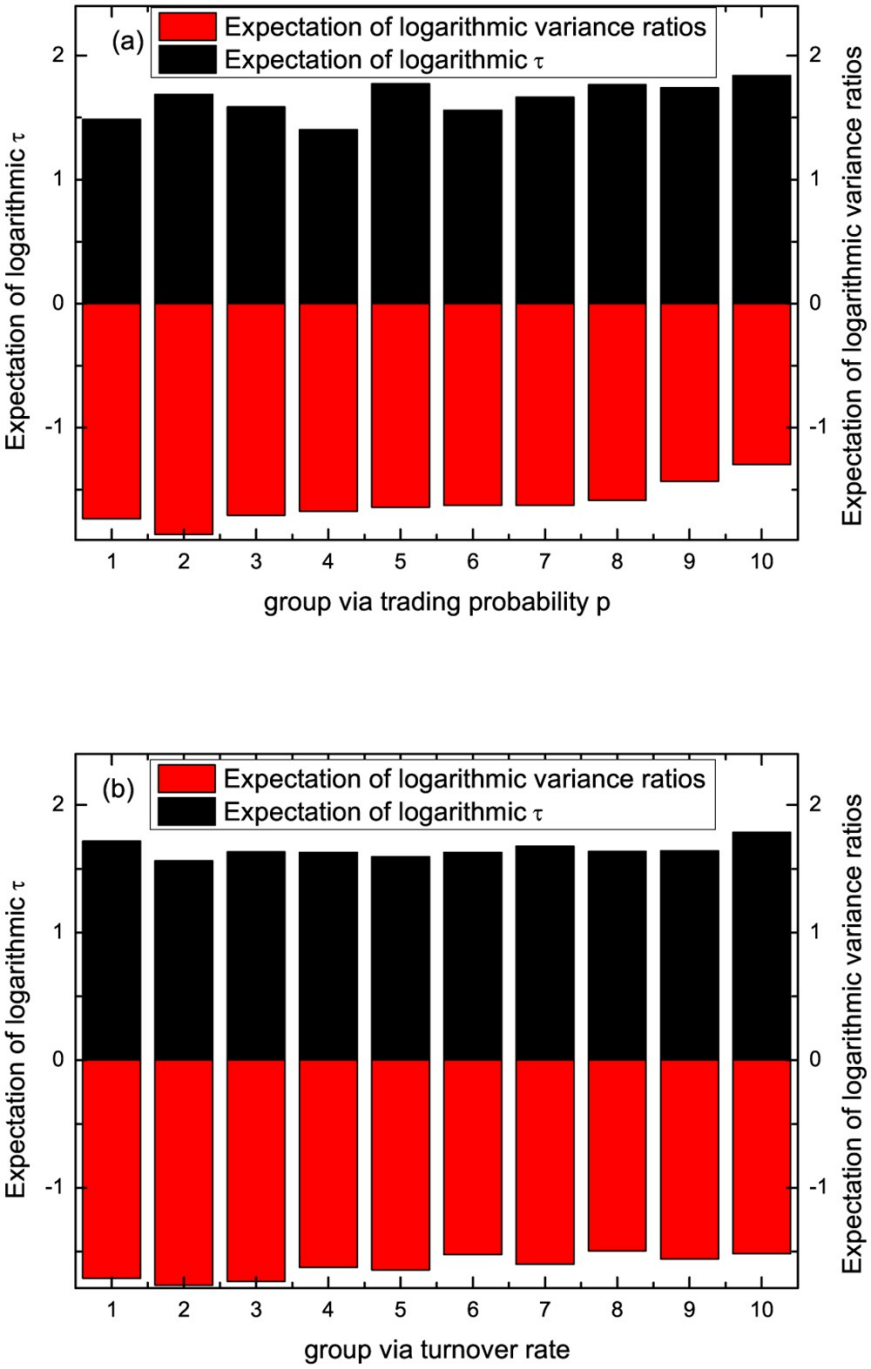
The expectation of efficiency indicator (delay time *τ* and variance ratios) versus group via trading probability *p* in (a) or via turnover rate in (b).

To explore the relationship between efficiency and liquidity, we calculate the delay time *τ* and variance ratios by our proposed methods and the liquidity measure trading probability *p* grouping and turnover rate as shown in Fig 8. After eliminating outliers based on variance ratios, 2340 stocks remain. We divide the A-share market stocks into ten groups according to the small to large trading probability *p* or turnover rate. Then expectations of delay time *τ* and variance ratios of efficiency measures in each group are calculated.

**Fig 9:**
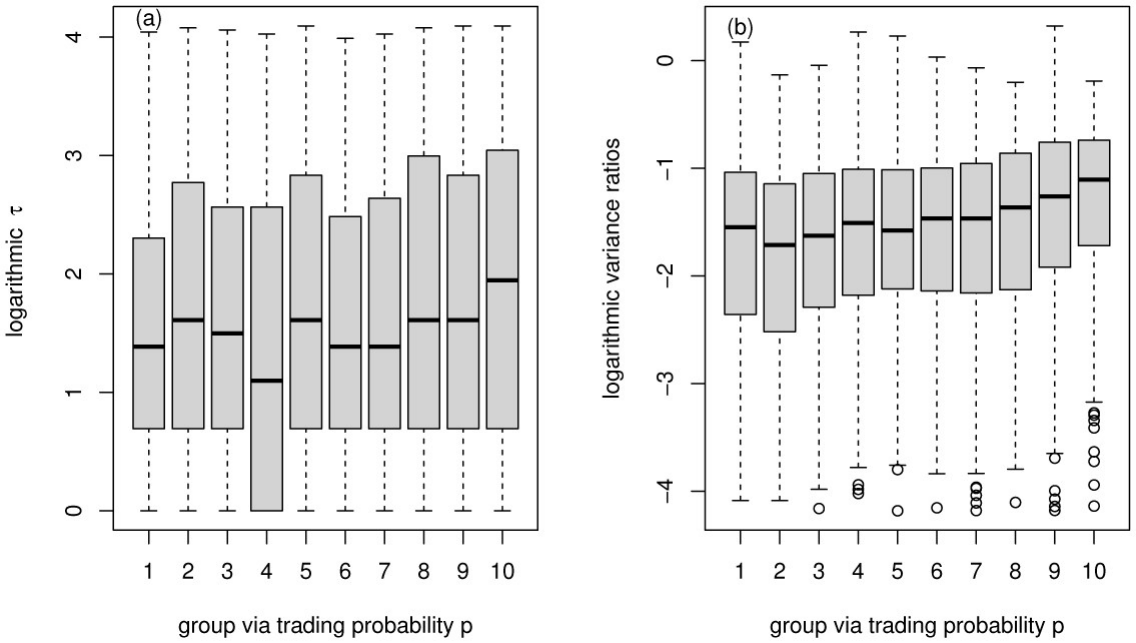
Boxplot of efficiency indicator (delay time *τ* and variance ratios) versus group via trading probability *p*.

**Fig 10:**
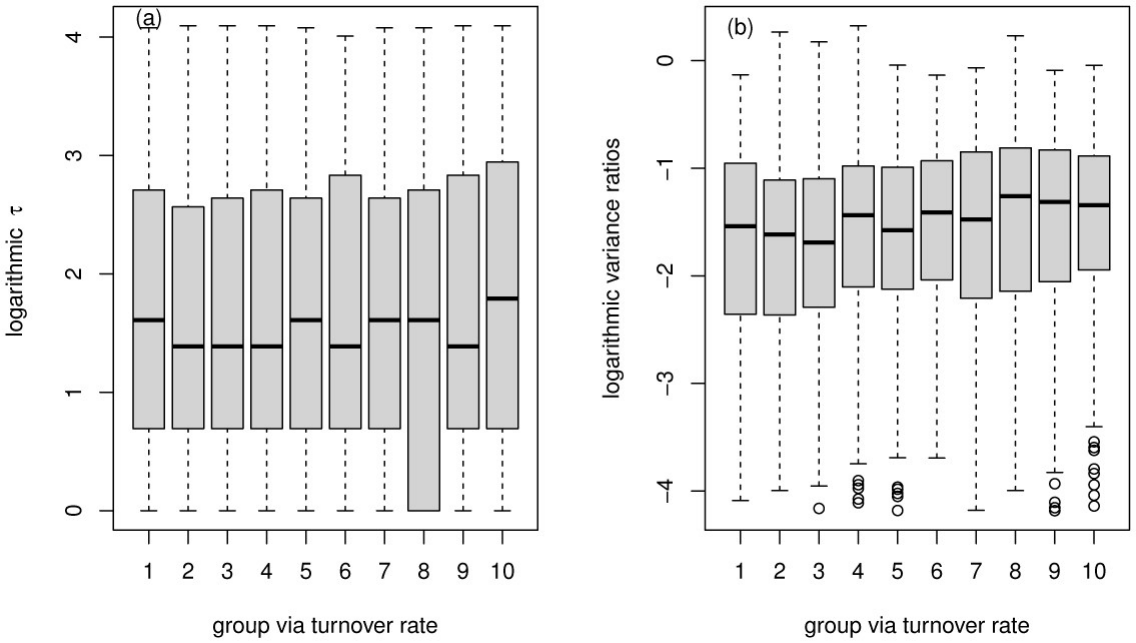
Boxplot of efficiency indicator (delay time *τ* and variance ratios) versus group via turnover rate.

We can see that: (1) the relationship between the two efficiency characteristics and the two liquidity characteristics all show non-monotonic characteristics; (2) there may be optimal liquidity that makes the market the most efficient. The result is consistent with many studies. When liquidity is low, the market is relatively inefficient; when liquidity is high, the market is rather hot, resulting in excessive speculation and the market efficiency showing not optimal. In Fig 8, delay time *τ* and liquidity characteristics are more consistent. The characteristics of the two efficiency measures obtained by the trading probability *p* grouping are consistent (Fig 8(b)). The two efficiency features obtained by the turnover rate grouping are relatively inconsistent (Fig 8(a)). To give a more detailed analysis, we present the box plot of the efficiency indicator (delay time *τ* and variance ratios) versus group via trading probability *p* in Fig 9, versus group via turnover rate in Fig 10. It can also be found that the delay time *τ* outpoise in the proposed model is much more than variance ratios, possibly because of the inaccuracy of *τ* obtained by using a simple DSSM estimation method.

## 6. Conclusions

The financial market is a complex system that has evolved due to the intricate behaviors of humans. As a crucial component of economic society, it enhances resource allocation efficiency. However, there are instances when markets may appear uneven, unstable, illiquid, and ineffective due to delayed impacts in information transmission. Therefore, we examine the roles of liquidity and delay in financial markets using our proposed optimal forecasting model. Drawing on previous research, we introduce a simplified stochastic delay model to establish delay time, conduct in-sample and out-of-sample forecasting performance comparisons, and then measure market efficiency through delay time to explore further the impact of market efficiency and liquidity on the market. Then the out-of-sample prediction performance is compared with the benchmark model ARIMA-GARCH. The differences and innovations of this paper are as follows. From the point of view of non-equilibrium statistical physics, the information delay is employed to measure market efficiency. The forecasting performance comparison is calculated by machine learning thinking and tested by the multi-loss function, SPA, AIC, and BIC methods. We propose six models for comparative analysis based on the likelihood estimation and Bayesian estimation methods. Combined with CSI 300 and S&P 500 index data, the prediction performance of six methods with and without delay is compared, and it is found that the prediction performance of the proposed delay model is better than that of the benchmark without delay model.

Additionally, using the suggested reduced stochastic delay model, we empirically compare the market’s efficiency and liquidity with information delay and trade probability. Regarding data, we select all A-shares of Shanghai and Shenzhen in the past five years to conduct research. The data time span is from January 1, 2014, to December 31, 2019. After excluding missing data, there are a total of 2,417 stocks. First, we use the simplest DSSM model based on the previously proposed method. Then combined with the likelihood function estimation, the estimated value of the proposed efficiency measurement *τ* and the transaction probability *p* are obtained. At the same time, it carries out an empirical comparative analysis with the efficiency measurement index variance ratios and the actual turnover rate in other documents. It can be found that: (1) The proposed efficiency measurement *τ* (the transaction probability *p*) is consistent with the comparison benchmark variance ratios (the turnover rate); (2) As the company size increases, expectations of *τ* and variance ratios both show an increasing characteristic, which also implies that the market efficiency of large market capitalization stocks may be lower than that of small market capitalization during this time period. (3) As the company size increases, the expectations of trading probability and turnover rate show decreasing characteristics. (4) The relationship between the two efficiency characteristics and the two liquidity characteristics shows non-monotonic characteristics, which means there may be optimal liquidity that makes the market most efficient. (5) Stochastic simulation shows that the increase in trading probability and delay time weakens the stability of return distribution and increases the risk. We discuss forecasting performances and measuring market efficiency via our proposed simplified stochastic delay model. The proposed theoretical methods and empirical results can provide suggestions for price prediction and evaluation of market efficiency and deliver new academic support for investment management and risk supervision.
